# All‐epiphyseal, all‐inside ACL reconstruction yields high return‐to‐sport and minimal growth‐related complications at mid‐ to long‐term follow‐up in skeletally immature patients

**DOI:** 10.1002/jeo2.70671

**Published:** 2026-02-26

**Authors:** Panagiotis Ntagiopoulos, Georgios Kalinterakis, Pierrenzo Pozzi, Dimitris Fligkos, George Themistocleous, Sotirios Themistokleous, Triantafyllia Dimou, Riccardo Compagnoni, Paolo Ferrua, Pietro Simone Randelli

**Affiliations:** ^1^ Hip and Knee Unit Mediterraneo Hospital Athens Greece; ^2^ U.O.C. 1° Clinica Ortopedica ASST G. Pini‐CTO Milan Italy; ^3^ Università degli Studi di Milano Milan Italy; ^4^ Department of Biomedical, Surgical and Dental Sciences Università Degli Studi di Milano Milan Italy; ^5^ Department of Biomedical Sciences for Health Università Degli Studi di Milano Milan Italy

**Keywords:** ACL reconstruction, all‐inside, knee, paediatric surgery, sport traumatology

## Abstract

**Purpose:**

Paediatric anterior cruciate ligament (ACL) injuries are becoming increasingly frequent. Surgical management remains challenging due to the risk of growth plate disturbance. Physeal‐sparing techniques aim to restore stability while minimizing this risk. The purpose of this study is to evaluate the clinical, functional and radiological outcomes of an all‐inside, all‐epiphyseal ACL reconstruction in skeletally immature patients, with particular attention to complications, return to sport (RTS) and the influence of associated injuries.

**Methods:**

Seventy‐eight patients (48 males and 30 females; mean age 13.1 years, range: 9–16) underwent physeal‐sparing ACL reconstruction between 2015 and 2024. All procedures were performed using hamstring autograft with all‐epiphyseal sockets under fluoroscopic guidance. Pre‐ and postoperative assessments included plain radiographs, magnetic resonance imaging (MRI) and clinical evaluation. Outcome measures were Lysholm, Paediatric International Knee Documentation Committee (Pedi‐IKDC) and visual analogue scale (VAS) scores, recorded preoperatively and at a mean follow‐up of 48 months (range: 24–82). Return‐to‐play rates, complications and growth disturbances were documented. Subgroup analyses investigated the effect of meniscal/chondral lesions, gender and surgical delay.

**Results:**

Significant postoperative improvements were achieved in all clinical scores: Lysholm (53.4–94.6, *p* < 0.01), Pedi‐IKDC (53.5–90.6, *p* < 0.01) and VAS (5.6–1.8, *p* < 0.01). No growth arrest, angular deformities, or leg length discrepancies were identified. Complications included one infection, two re‐ruptures and one residual laxity. RTS was achieved in 94.8% of patients, with a mean recovery period of 11.1 months. Meniscal and chondral lesions were associated with inferior outcomes and delayed return‐to‐play. Delayed surgery significantly increased the risk of meniscal injury (odds ratio [OR]: 5.1, *p* = 0.001).

**Conclusion:**

All‐inside physeal‐sparing ACL reconstruction demonstrates excellent mid‐ to long‐term outcomes in skeletally immature patients. The technique offers high RTS rates and minimal growth‐related complications. Early surgical intervention may reduce the risk of secondary meniscal and chondral damage.

**Level of Evidence:**

Level IV, retrospective case series.

**Abbreviations**
ACL: anterior cruciate ligament
GLM: general linear model
IKDC: International Knee Documentation Committee
IOC: International Olympic Committee
MRI: magnetic resonance imaging
PROMs: patient‐reported outcome measures
RTS: return to sport
VAS: visual analogue scale

## INTRODUCTION

Anterior cruciate ligament (ACL) tears in skeletally immature patients have increased substantially in recent years. Population‐based studies from Australia and Finland have reported a two‐ to threefold rise in paediatric ACL injuries over the past decade. This trend is attributed not only to advances in imaging and heightened diagnostic awareness but also to changes in youth sports participation, including earlier specialization, intensive training programs and year‐round athletic activity [10, 20, 30, 33].

Historically, nonoperative management or delayed ACL reconstruction until skeletal maturity was advocated to minimize the risk of growth disturbance. However, these strategies have been associated with persistent knee instability, reduced return‐to‐sport (RTS) rates and a significantly increased risk of secondary meniscal and chondral injury, particularly in active children [23, 26, 31]. As a result, early surgical stabilization usingesult, early surgical stabilization usin techniques that respect the physes has gained increasing acceptance.

Among physeal‐sparing options, all‐epiphyseal ACL reconstruction has emerged as a favoured approach because it allows tunnel placement entirely within the epiphysis, thereby avoiding direct physeal violation [21, 24, 29]. Biomechanical studies have shown that these techniques can restore near‐native knee kinematics and reduce abnormal joint loading [17, 25]. Several all‐epiphyseal methods have been described, including the Anderson, Ganley–Lawrence and McCarthy techniques, which differ mainly in tunnel orientation and fixation strategy [1, 9, 21, 24]. Although generally considered safe, rare growth‐related complications such as lateral distal femoral physeal closure or limb overgrowth have been reported, potentially related to thermal injury, periosteal disruption or altered local vascularity [18, 22, 33].

Alternative physeal‐sparing reconstructions, such as iliotibial band or over‐the‐top techniques, may still be indicated in selected very young patients with substantial growth remaining. However, these procedures are technically demanding and do not fully restore anatomic ACL function [3, 8, 17, 19, 35]. Overall, mid‐ to long‐term outcome data for physeal‐sparing ACL reconstruction remain limited, and substantial heterogeneity persists in reported functional outcomes and paediatric patient‐reported outcome measures (PROMs) [6, 32, 33, 34].

In parallel, lateral extra‐articular procedures, including lateral extra‐articular tenodesis (LET) and anterolateral ligament (ALL) reconstruction, have attracted renewed interest as adjuncts to ACL reconstruction for improving rotational stability and reducing graft failure, particularly in high‐risk adolescents [12]. Nevertheless, current American Academy of Orthopaedic Surgeons (AAOS) guidelines provide only a moderate recommendation for their use, reflecting limited long‐term evidence, while most supporting studies involve skeletally mature or nearly mature patients [5]. Paediatric‐specific data remain sparse and heterogeneous, with published series typically characterized by small sample sizes and short‐term follow‐up [7, 28].

In this study, we report the mid‐ to long‐term outcomes of a McCarthy‐type all‐epiphyseal ACL reconstruction technique, based on the principles described by McCarthy et al., which has demonstrated favourable biomechanical characteristics and magnetic resonance imaging (MRI)‐based physeal safety in early investigations [27]. Therefore, the purpose of the present study was to assess the clinical, functional and radiological outcomes of this all‐epiphyseal ACL reconstruction technique in skeletally immature patients. We hypothesized that it would provide satisfactory knee stability, high RTS rates and minimal growth‐related complications across the paediatric age spectrum, regardless of concomitant meniscal or chondral pathology.

## MATERIAL AND METHODS

This retrospective study was conducted between September 2015 and June 2024. During this period, 78 patients aged 9–16 years underwent surgery for ACL rupture using an all‐inside, all‐epiphyseal technique. All procedures were performed at the same hospital by a senior surgeon (P.G.N.), following approval from the institution's scientific research board (54/20‐06‐2023). Informed consent was obtained from all patients and their parents prior to participation.

### Patient selection

The diagnosis of ACL rupture was made in all cases through clinical examination and MRI scans. In addition to MRI, the preoperative imaging protocol included anteroposterior (AP) and lateral radiographs. Limb length and alignment were evaluated through clinical examination. In cases of suspected deformity, long‐leg standing radiographs were obtained to allow for accurate quantification. Patients included in this case series met the following criteria: (1) skeletal immaturity, (2) primary ACL reconstruction using the all‐inside, all‐epiphyseal technique, (3) concomitant meniscus and chondral injuries, (4) use of a hamstring autograft and (5) at least 24 months of follow‐up.

Exclusion criteria were: (1) closed physis, (2) prior knee procedures or concomitant injury to other knee ligaments and (3) patients with underlying syndromes.

### Demographics and clinical assessment

A retrospective analysis of our database was conducted. Demographic information and all relevant data points were collected from two senior surgeons D.F. and G.K.: age, gender, concomitant meniscal and chondral injuries, time to surgery, the follow‐up duration and the return to play. Preoperative and postoperative clinical and functional evaluations of the patients were conducted using the visual analogue scale (VAS), the Lysholm Knee Scoring System and the Paediatric International Knee Documentation Committee (Pedi‐IKDC). We compared these scores preoperatively and in the last follow‐up.

Anterior knee laxity and rotational stability were assessed clinically. The Lachman test was evaluated qualitatively and categorized as firm endpoint, soft endpoint, clearly increased anterior translation and grossly unstable. The pivot‐shift test was similarly graded as absent, glide, clunk and grossly positive. Examinations were performed by two senior orthopaedic examiners, and both preoperative and postoperative findings were recorded.

Limb length was assessed clinically using a standardized tape‐measure method from the anterior superior iliac spine (ASIS) to the medial malleolus. Lower limb alignment was evaluated through standing clinical inspection, comparing bilateral lower‐limb axes and observing for varus/valgus asymmetry. These measurements were performed preoperatively and repeated at the final follow‐up visit. Long‐leg radiographs were obtained only when clinical examination raised suspicion of limb‐length discrepancy or angular deformity, to avoid unnecessary radiation exposure in asymptomatic skeletally immature patients. Skeletal maturity was estimated based on chronological age and MRI assessment of physeal status. Neither Tanner staging nor atlas‐based bone age methods were used due to the retrospective nature of the study.

### MRI protocol and physeal assessment

All MRI studies were performed on a 3.0‐T scanner using a dedicated knee coil. The protocol included sagittal and coronal proton‐density fat‐suppressed sequences, sagittal T1‐weighted images and axial T2‐weighted images, with a slice thickness of 3 mm. Physeal evaluation followed widely accepted paediatric MRI criteria, assessing growth‐plate signal characteristics and continuity to classify physes as open, closing or closed [30]. Each physis was examined for focal irregularity, asymmetric closure or evidence of bar formation. In addition, graft signal intensity, tunnel position and tunnel–graft integration were qualitatively evaluated on all sequences.

### Surgical technique

**Figure 1:**
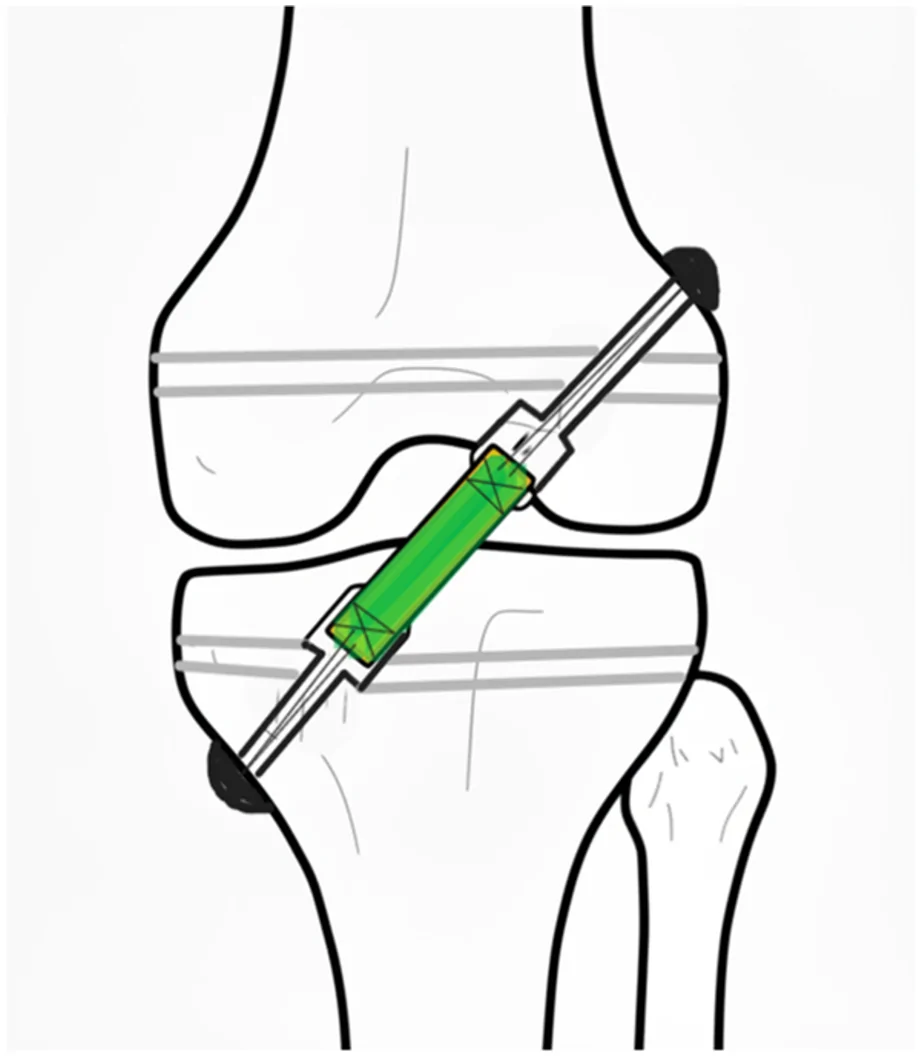
Schematic drawing of the all‐epiphyseal all‐inside surgical technique described in the article.

The surgical technique used in this series follows the principles of all‐epiphyseal, physeal‐sparing ACL reconstruction described in the literature (Figure 1) [24].

All operations were performed by a single senior surgeon (P.G.N.). For all patients, a hamstring autograft, specifically the semitendinosus, was used. Graft harvesting was performed in a standard fashion. If the graft diameter was insufficient (<7–8 mm), the gracilis was also harvested. Graft preparation was done by the senior surgeon (D.F.) in the ancillary table. In parallel, the lead surgeon performed a standard diagnostic knee arthroscopy. All chondral and meniscal pathology was appropriately addressed. The ACL tear was then identified and debrided to expose the tibial and femoral insertional footprints. Next, the all‐epiphyseal sockets were created under fluoroscopic guidance. The femoral socket was prepared first using an outside‐in femoral guide through the anterolateral portal at 90°–95°. The guide was routinely positioned at the centre of the femoral footprint, approximately 2–3 mm from the posterior wall of the femoral condyle. For the tibial socket, the tibial ACL guide was set at 55° and positioned at the centre of the tibial footprint. A specialized retro cutter reamer was used for both tunnels to create blind (all‐inside) sockets, each measuring 20–25 mm. Fixation was achieved using adjustable‐loop suspensory devices. With the knee in full extension, the graft was visualized arthroscopically, and both the femoral and tibial adjustable‐loop constructs were progressively tensioned until stable fixation was confirmed.

### Postoperative evaluation and rehabilitation

All patients were encouraged to begin range of motion exercises as soon as possible after surgery. During the first week, they were allowed weight‐bearing as tolerated with crutches. Aggressive ice therapy was recommended for swelling control. Formal physical therapy was initiated one week postoperatively. Phase 1 (approximately 4–6 weeks) focused on reducing swelling, restoring range of motion and achieving early quadriceps activation. Phase 2 (6 weeks to 3 months) emphasized progressive strengthening exercises, neuromuscular training and balance work, gradually increasing intensity while avoiding excessive stress on the healing graft. Return to running and sport‐specific drills was introduced gradually after 4–6 months. Clearance for return to competitive sports, typically after 9–12 months, was based on functional assessment, including single‐leg hop testing, evaluation of functional movement patterns (such as single‐leg squatting and landing mechanics), restoration of full range of motion, and the absence of pain, effusion or instability.

Close monitoring and individualized progression were essential to optimize outcomes and minimize reinjury risk. The patients were routinely evaluated clinically at the following intervals: 1 week, 2 weeks, 1 month, 3 months, 6 months, 9 months, 12 months and then annually until skeletal maturity. Immediately after surgery, AP and lateral radiographs were obtained, while MRI scans were performed at 6 months and 1.5 years postoperatively to exclude any potential growth plate disturbance.

### Statistical analysis

Descriptive statistics were used to summarize patient demographics and clinical characteristics. Continuous variables were presented as means and standard deviations or medians, while categorical variables were expressed as frequencies and percentages. Paired‐sample *t* tests were performed to compare preoperative and postoperative PROMs, including the Lysholm score, IKDC subjective score and visual analogue scale (VAS) for pain. To evaluate the influence of clinical factors on postoperative outcomes, a general linear model (GLM) was applied, with postoperative PROMs as dependent variables. Independent variables included meniscal tear, chondral damage, gender and age. A binary logistic regression analysis was conducted to examine whether the time interval from injury to surgery was associated with the presence of meniscal injury. Due to the limited number of chondral damage events (*n* = 6), a Fisher's exact test was used to assess the association between surgical delay and the presence of chondral lesions. A *p* value of <0.05 was considered statistically significant. All analyses were performed using SPSS software (version 29; IBM Corp.).

## RESULTS

### Patient characteristics

Seventy‐eight adolescents (mean age 13.06 years, range: 9–16) underwent all‐epiphyseal ACL reconstruction. The cohort consisted of 48 males and 30 females. The mean injury‐to‐surgery interval was 41.46 days (range: 2–240). Meniscal pathology was identified in 44.9% of patients, with chondral lesions present in 7.7%. Among the 35 meniscal tears treated, 17 involved the medial meniscus, 13 the lateral meniscus and 5 were combined injuries. The majority of tears were managed with all‐inside meniscal repair, whereas non‐repairable lesions were treated with partial meniscectomy. When chondral lesions were encountered, they were predominantly low‐grade (Outerbridge I–II) and were addressed with arthroscopic debridement. The mean return‐to‐play interval was 11.09 months (range: 9–18). Postoperative complications included one superficial infection, two graft re‐ruptures and one case of residual laxity, defined as clearly increased anterior translation on Lachman testing. A summary of patient characteristics is provided in Table 1.

**Table 1:** Patients' demographic and complications.

Age, mean (range)	13.06 (9–16)
Number of girls	30
Number of boys	48
Meniscal pathology (%)	44.9%
Chondral injury (%)	7.7%
Complications, number of patients	
Infection	1
Re‐rupture	2
Residual laxity	1
Time between the initial trauma and the surgical intervention, mean (range), days	41.46 days (2–240)
Return‐to‐play, mean (range), months	11.09 (9–18)

### Clinical outcomes

Significant postoperative improvement was observed across all clinical outcome measures. Mean Pedi‐IKDC increased from 53.53 to 90.61, Lysholm score from 53.42 to 94.62, and VAS decreased from 5.62 to 1.79 (all *p* < 0.01). These changes are presented in Table 2. At final follow‐up, Lachman testing demonstrated a firm endpoint in all patients except one, who showed clearly increased anterior translation. Pivot‐shift testing was negative in all patients. Range of motion was fully restored in the majority of cases; six patients had a mild extension deficit (<5°), and one patient exhibited a mild flexion deficit at final follow‐up.

**Table 2:** Preoperative and postoperative, Pedi‐IKDC, Lysholm and VAS scores.

Score	Preoperative	Postoperative	*t* Value
	*M* (SD)	*M* (SD)	
VAS	5.62 (1.21)	1.79 (1.02)	35.51*
Lysholm	53.42 (9.19)	94.62 (5.32)	−44.05*
Pedi‐IKDC	53.53 (11.23)	90.61 (6.06)	−30.01*

Abbreviations: *M*, mean; Pedi‐IKDC, Paediatric International Knee Documentation Committee; SD, standard deviation; VAS, visual analogue scale.

* *p* significant at < 0.01 (*t* test).

### Meniscal and chondral injury: Effect on outcomes and association with surgical timing

Adolescents with a concomitant meniscal tear demonstrated significantly worse postoperative functional outcomes compared with those without meniscal injury. Adjusted mean scores were lower in the meniscal‐injury group for both Pedi‐IKDC and Lysholm, and return‐to‐play time was longer, whereas VAS scores were slightly higher but not significantly different. These differences are detailed in Table 3.

**Table 3:** Comparison of postoperative outcomes between adolescents with and without meniscal injury (general linear model results).

Outcome	Meniscal injury	No meniscal injury	Mean difference	*F* (df1, df2)	Partial *η* ^2^
	*M* (SD)	*M* (SD)			
Pedi‐IKDC	85.81 (5.17)	94.51 (3.33)	−8.38*	*F* (1, 73) = 67.36	0.48
Lysholm	91.31 (5.06)	97.30 (3.84)	−5.09*	*F* (1, 73) = 22.95	0.23
VAS	2.03 (1.04)	1.60 (0.97)	−0.35	*F* (1, 73) = 2.01	0.027
RTP (months)	12.45 (1.56)	10.12 (1.40)	2.33*	*F* (1, 69) = 2.01	0.36

Abbreviations: *M*, mean; Pedi‐IKDC, Paediatric International Knee Documentation Committee; RTP, return to play; SD, standard deviation; VAS, visual analogue scale.

* *p* significant at < 0.001.

Chondral injury was associated with significantly lower postoperative Pedi‐IKDC scores—*F*(1, 67) = 16.05, *p* < 0.001, *η* ^2^ = 0.193—and delayed return to play—*F*(1, 63) = 16.57, *p* < 0.001, *η* ^2^ = 0.208). No significant differences were observed in Lysholm (*p* = 0.343) or VAS scores (*p* = 0.166).

A binary logistic regression analysis demonstrated that a longer injury‐to‐surgery interval was significantly associated with the presence of a meniscal tear at arthroscopy. Each additional month of delay increased the odds of meniscal damage by more than five‐fold (odds ratio [OR] = 5.131, 95% confidence interval [CI] [1.88–13.96], *p* = 0.001). The model demonstrated good fit (Hosmer–Lemeshow *p* = 0.784). Due to the small number of chondral injury events (*n* = 6), logistic regression was not performed. Instead, a Fisher's exact test was used. Patients with chondral damage had significantly longer surgical delay compared to those without (median 6.1 vs. 0.7 months, *p* < 0.001).

### Effect of gender and age

Gender showed a significant association with VAS—*F*(1, 67) = 5.19, *p* = 0.026, *η* ^2^ = 0.072—and return‐to‐play duration—*F*(1, 63) = 5.11, *p* = 0.027, *η* ^2^ = 0.075)—with female patients reporting slightly higher pain scores and longer recovery times. No significant effect of gender or age was detected for Pedi‐IKDC or Lysholm scores. Age was not significantly associated with any postoperative outcome.

## DISCUSSION

The main finding of this study is that early ACL reconstruction using an all‐inside, physeal‐sparing technique in skeletally immature patients is a safe and effective option, resulting in marked improvement in PROMs and VAS scores. Our analysis also showed that meniscal and chondral injuries were associated with less favourable outcomes and occurred more frequently in patients who experienced a longer delay before surgery.

Managing ACL injuries in children remains challenging because of clinical uncertainty and limited high‐quality evidence. Decisions regarding nonoperative versus surgical treatment, technique selection and timing of reconstruction continue to be debated. In response, the International Olympic Committee (IOC) issued a consensus statement in 2018, and a subsequent European Society of Sports Traumatology (ESSKA) survey evaluated how widely these recommendations are followed in clinical practice [4].

Although the principles of ACL reconstruction are similar in children and adults, the presence of open physes in skeletally immature patients requires specific surgical modifications to avoid growth‐plate injury. This consideration has led to the development of all‐epiphyseal techniques designed to minimize physeal risk. A recent systematic review by Migliorini et al. reported that all‐epiphyseal reconstruction offers faster and higher RTS rates than trans‐epiphyseal methods, with no significant differences in complications or functional outcomes [26]. Wong et al., in a narrative review, found that all‐epiphyseal techniques were associated with a higher incidence of limb overgrowth, whereas the over‐the‐top technique showed a greater rate of angular deformities, although both approaches demonstrated comparable rates of graft re‐rupture [35].

In this study, Pedi‐IKDC, Lysholm and VAS scores were used to assess patients' perceived knee function and overall treatment outcome. All measures showed significant postoperative improvement. Notably, patients with concomitant meniscal tears at the time of surgery reported lower functional scores than those without meniscal injury, indicating a measurable impact of associated pathology on recovery. Our analysis also demonstrated a clear association between surgical timing and the likelihood of meniscal damage; specifically, each additional month of delay increased the odds of a meniscal tear by approximately fivefold. This finding aligns with results from Kolin et al., who observed a 2% increased risk of medial meniscal injury for each week of delayed surgical stabilization [20]. Similarly, a recent systematic review reported that postponing ACL reconstruction in paediatric and adolescent patients by more than 12 weeks increased the risk of meniscal injury by more than fourfold [14].

The incidence of chondral lesions in our cohort was lower than that of meniscal injuries, consistent with previous reports [2]. However, because meniscal and chondral pathology frequently coexist, surgeons must maintain a high index of suspicion during arthroscopy, particularly within the same compartment [11]. In our series, chondral injuries were less common than meniscal tears, occurring in 7.7% of patients. Most lesions were Grade 1 or 2 and required only minimal debridement. Consistent with existing literature, our findings showed that chondral damage was more frequently observed in patients who experienced a longer delay before ACL reconstruction [16].

Physeal‐sparing ACL reconstruction has been associated with higher RTS rates than transphyseal techniques [15]. A prospective cohort study demonstrated that patients undergoing all‐epiphyseal ACL reconstruction achieved superior quadriceps symmetry and knee‐related function compared with those treated transphyseally, which may help explain these differences [13]. In our cohort, 94.8% of patients RTS at an average of 11.09 months. Because early RTS, young age and participation in pivoting sports increase the risk of graft re‐tear, a delayed RTS timeline is recommended [4]. We observed two re‐ruptures (2.5%), supporting the practice of allowing RTS no earlier than 9–12 months postoperatively, with active parental involvement in rehabilitation.

### Limitations

This study has several limitations that should be acknowledged. First, its retrospective design may have introduced information and selection bias, potentially affecting the completeness and accuracy of the collected data. Second, the absence of a control group limits the ability to establish causal relationships or directly compare outcomes between treatment strategies. Skeletal maturity was assessed using chronological age and MRI evaluation of physeal status, but formal maturity assessments such as Tanner staging or atlas‐based bone‐age methods were not performed. Given the broad age range of the cohort, differences in remaining growth potential could not be quantified precisely and may have influenced growth‐related risk assessment. Similarly, limb length and alignment were evaluated clinically, and long‐leg standing radiographs were obtained only when clinically indicated; therefore, subtle or asymptomatic alignment abnormalities may have been underestimated.

Postoperative knee stability was assessed primarily through qualitative clinical examination, including Lachman and pivot‐shift testing. The lack of standardized instrumental measurements (e.g., arthrometric evaluation) represents a limitation in the objective quantification of residual laxity. In addition, although validated PROMs were used, the study did not include objective functional assessments such as strength testing or biomechanical performance measures. Regarding associated intra‐articular pathology, meniscal and chondral injuries were recorded based on arthroscopic findings. While this represents the diagnostic gold standard, the study design did not allow reliable differentiation between lesions present but not clearly visualized on preoperative MRI and those that may have developed or progressed during the injury‐to‐surgery interval. As such, analyses relating surgical delay to meniscal pathology should be interpreted as associations rather than evidence of lesion progression.

## CONCLUSION

All‐inside physeal‐sparing ACL reconstruction demonstrates excellent mid‐ to long‐term outcomes in skeletally immature patients. The technique offers high RTS rates and minimal growth‐related complications. Early surgical intervention may help to reduce the risk of secondary meniscal and chondral damage.

## AUTHOR CONTRIBUTIONS

*Conception of the study*: Panagiotis Ntagiopoulos, Paolo Ferrua, Pierrenzo Pozzi and Georgios Kalinterakis. *Acquisition of data:* Panagiotis Ntagiopoulos and Georgios Kalinterakis. *Analysis of data*: Panagiotis Ntagiopoulos and Georgios Kalinterakis. *Drafting of the work*: Pierrenzo Pozzi, Paolo Ferrua, Panagiotis Ntagiopoulos, George Themistocleous, Sotirios Themistokleous and Georgios Kalinterakis. *Revising it clinically for important intellectual content:* Panagiotis Ntagiopoulos, Paolo Ferrua, Riccardo Compagnoni, Pietro Simone Randelli, Triantafyllia Dimou, George Themistocleous, Sotirios Themistokleous and Dimitris Fligkos. *Final approval of the version to be published*: Pierrenzo Pozzi, Georgios Kalinterakis, Panagiotis Ntagiopoulos, Paolo Ferrua, Riccardo Compagnoni, Pietro Simone Randelli, Triantafyllia Dimou and Dimitris Fligkos.

## CONFLICT OF INTEREST STATEMENT

The authors declare no conflicts of interest.

## ETHICS STATEMENT

This study was approved by the ethical committee of Mediterraneo Hospital, Athens, Greece (number: 54/20‐06‐2023).

## Data Availability

Data outside the one reported in the manuscript are available upon request from the authors.
